# Kidney Allograft Cyst Infection

**DOI:** 10.1016/j.ekir.2020.04.013

**Published:** 2020-04-28

**Authors:** Geraldo Rubens Ramos de Freitas, Stan Benjamens, Priscila Dias Gonçalves, Maria Leticia Cascelli de Azevedo, Evandro Reis da Silva Filho, José Osmar Medina-Pestana, Robert A. Pol, Thiago Reis

**Affiliations:** 1 Department of Nephrology and Kidney Transplantation, Clínica de Doenças Renais de Brasília (CDRB), Brasília, Brazil; 2 Department of Surgery, Division of Transplantation Surgery, University Medical Center Groningen, University of Groningen, Groningen, Netherlands; 3 Medical Imaging Center, University Medical Center Groningen, University of Groningen, Groningen, Netherlands; 4 Department of Nephrology, University Hospital of Brasilia (HUB), Brasília, Brazil; 5 Department of Nephrology, Hospital do Rim e Hipertensão, Federal University of São Paulo, São Paulo, Brazil

## Introduction

Because of the persistent shortage of kidney allografts available for transplantation, living donor selection criteria have been expanded in recent years. As an example, kidney cysts found during predonation imaging screening are no longer a contraindication to living donor kidney donation.^1^^,^^2^

Kidney cysts are characterized under the Bosniak classification system.^3^ The most common is the Bosniak grade 1, which is of benign morphology and usually referred to as a simple cyst. As part of the predonation screening, potential living kidney donors undergo contrast-enhanced computed tomography (CT) imaging. This predonation imaging screening reveals asymptomatic and usually clinically nonrelevant anatomical deviations.^4^ The literature available on this topic suggests that simple cysts are found in approximately 10% of living donors.^5^^,^^6^ The prevalence of simple cysts increases progressively with age, and the majority of them remain asymptomatic.^7^^,^^8^ Even though kidney allografts with simple cysts have been shown to be associated with reduced transplant function, transplantation of kidney allografts with simple cysts is commonly performed.^5^ Cyst unroofing or excision of the cyst is generally not necessary; however, it can be performed if the cyst is large and superficial.^6^

The case reported here highlights a potential clinical challenge related to living donor kidney transplantation of a kidney allograft with a simple cyst and shows that recurrent cyst infections should be considered as part of the differential diagnosis of fever in kidney transplant patients.

## Case Presentation

**Figure 1:**
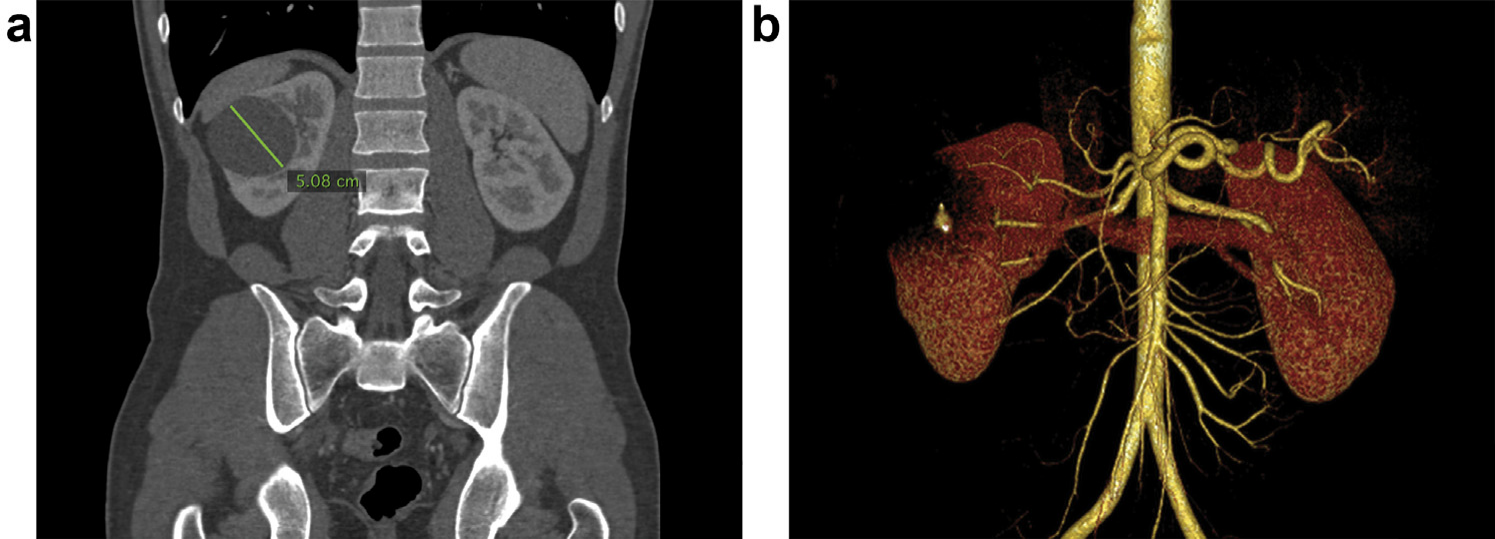
Computed tomography images of the donor, showing (a) a 5.08-cm renal cyst, with (b) an imperceptible wall and benign appearance, classified as Bosniak grade 1.

A 34-year-old woman with chronic kidney disease due to diabetic nephropathy received a preemptive living donor kidney transplant from her healthy 32-year old husband. During predonation screening, a cyst was identified in the right kidney on contrast-enhanced CT. This 5.08-cm cyst had an imperceptible wall, was round, had a benign appearance, and was classified as a Bosniak grade 1 kidney cyst (Figure 1). Mismatch results for human leukocyte antigen (HLA) showed 1, 2, and 1 mismatch for HLA-A, -B, and -DR antigens, respectively. Pretransplantation panel reactive antibody (PRA) for both class I and II was 0. After discussion at our multidisciplinary conference, the transplantation was cleared to proceed despite the renal cyst. This was based on the recommendations in the Kidney Disease: Improving Global Outcomes Clinical Practice Guidelines on the Evaluation and Care of Living Kidney Donors.^1^ The cyst was unroofed following donor nephrectomy, after which the transplantation procedure was performed. The donor’s right kidney was implanted in the recipient’s right iliac fossa, and the postoperative period was uncomplicated for both donor and recipient. The induction regimen included thymoglobulin 3 mg/kg single dose and methylprednisolone 1000 mg. The patient was on tacrolimus, azathioprine, and prednisone for maintenance therapy. During the first year of post-transplantation follow-up, there were no transplant-related complications, and the kidney allograft function remained stable.

**Figure 2:**
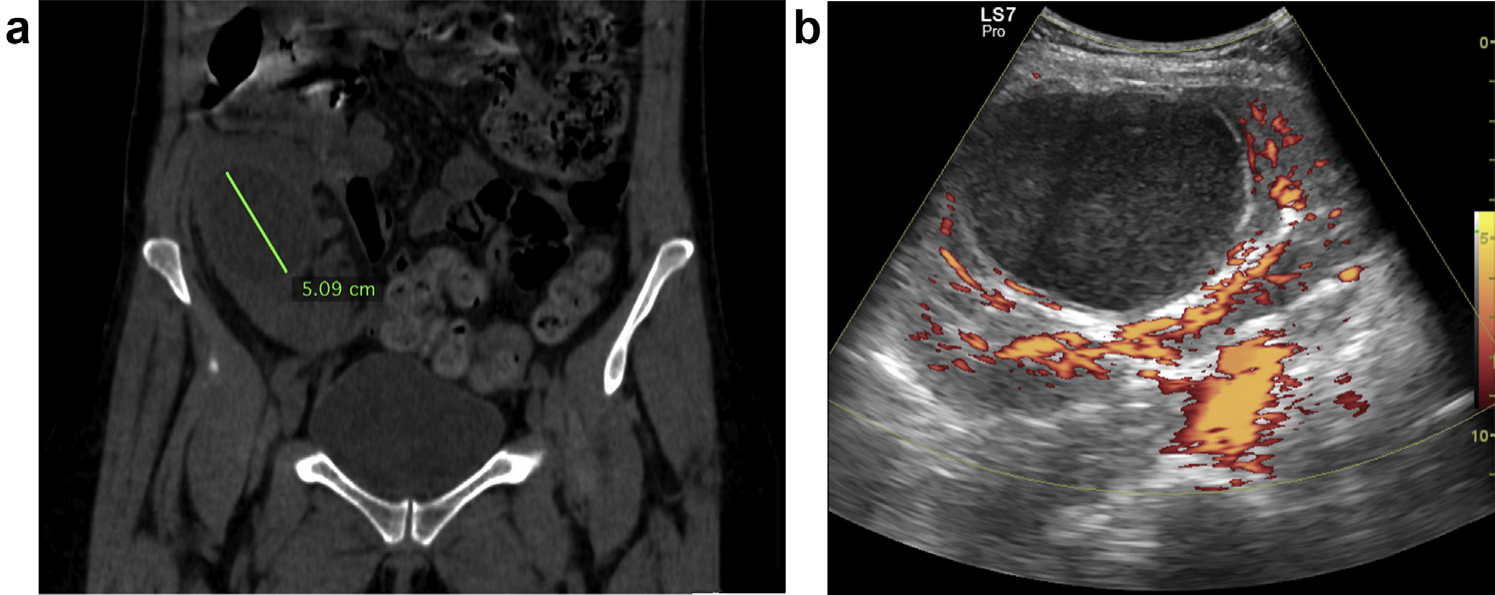
(a) Computed tomography image of the transplant recipient at 1 year after transplantation, showing the initially unroofed cyst, which regained full size due to allograft encapsulation, and (b) an ultrasound of the kidney allograft with signs of simple cyst infection.

Fourteen months after transplantation, the transplant recipient presented with pain in the right iliac fossa, without fever or signs of a urinary infection. Laboratory tests were unremarkable, and plasma beta-human chorionic gonadotropin was negative. An abdominal computed tomogram did not reveal the source of the symptoms. However, the kidney allograft showed a recurrence of the previously unroofed simple cyst (Figure 2). Without a clear diagnosis regarding the lower abdominal pain, analgesics were prescribed (i.e., paracetamol 1 g 4 times per day, and metamizole [dipyrone] 1 g up to 4 times per day), which provided adequate pain relief, and the patient was subsequently discharged.

Six days after discharge, the patient presented again with worsening pain and fever (39 °C) but without any urinary symptoms. Laboratory testing revealed leukocytosis with left shift, acute kidney injury stage 1,^9^ normal urine analysis results, and negative urine culture (Table 1).^9^ Doppler ultrasound of the kidney allograft showed normal perfusion; however, heterogeneity and debris in the simple cyst was observed (Figure 2), leading to the possibility that a cyst infection was present.

**Table 1:** Main biochemical data from day of admission

Test	Range	Results		
		Day 1	Day 4	Day 21
Hemoglobin (g/dl)	13–17	10.1	8.8	11
Platelets (/mm^3^)	150,000–400,000	495,000	581,000	358,000
Leucocytes (/mm^3^)	4000–10,000	10,500	13,300	7500
CRP (mg/l)	<10		154.62	2.52
Creatinine (mg/dl)	0.6–1.2	1.85	1.58	1.37
Urea (mg/dl)	7–20	35	21	50
Sodium (mEq/l)	135–145	125	132	139
Potassium (mEq/l)	3.5–5.0	4.9	4.7	4.8
Protein, urine	—	NEG	NEG	
Nitrite, urine	—	NEG	NEG	
Urine culture	—	NEG		
Blood culture	—	NEG		

CRP, C-reactive protein; NEG, negative.

**Figure 3:**
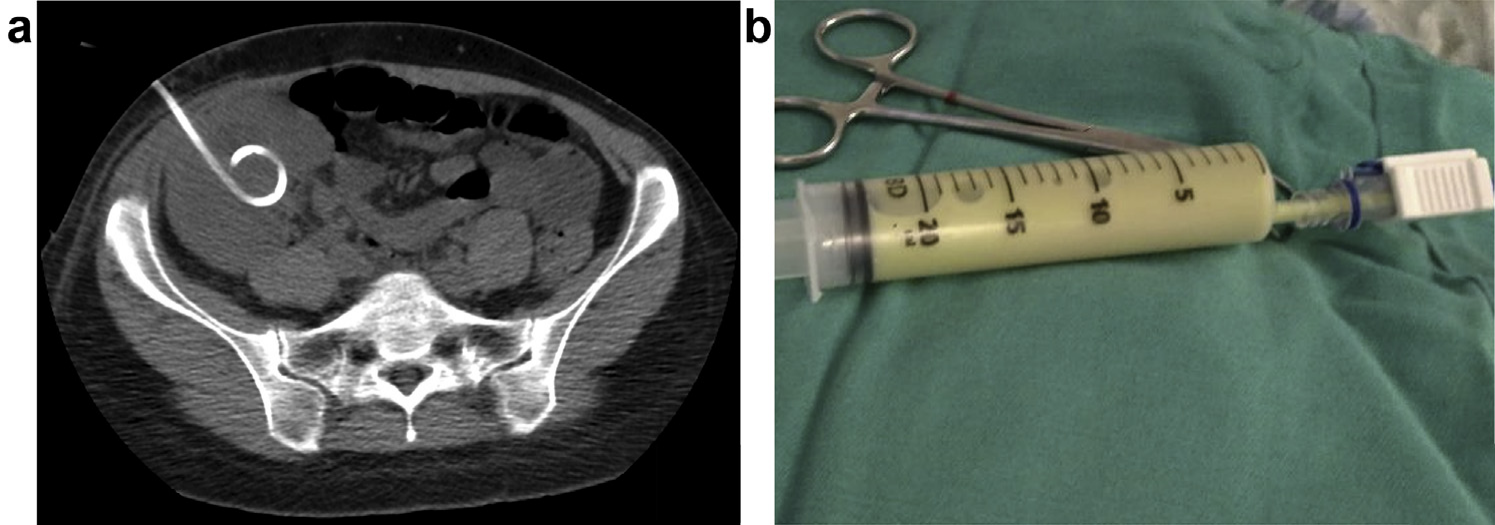
Percutaneous computed tomography−guided drainage of (a) the simple cyst with (b) purulent secretion.

**Figure 4:**
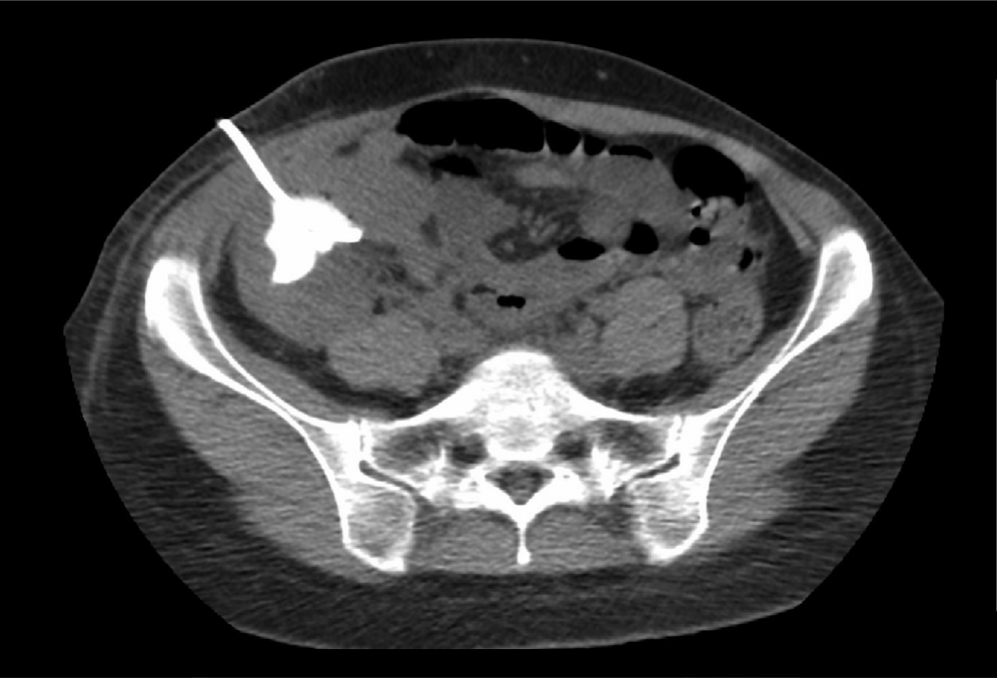
Contrast-enhanced computed tomography study showing no connection between cyst and urinary tract.

Empirical treatment with intravenous ciprofloxacin 400 mg 2 times per day was initiated. A partial clinical response on this antibiotic therapy was observed: the patient’s fever lasted for 48 hours, after which her body temperature normalized. Sustained leukocytosis was observed for 3 days after therapy initiation. Pain relief and reduction of asthenia was initially achieved in 24 hours, but both returned after 72 hours. A percutaneous CT-guided drainage of the infected cyst resulted in removal of 170 ml of purulent secretion. A pigtail drainage catheter was left in the cyst (Figure 3). Contrast-enhanced CT showed no connection of the cyst with the urinary tract (Figure 4). Serum creatinine was 1.53 mg/dl and cyst creatinine was 1.41 mg/dl. Analysis of the cyst fluid revealed 656,000 cells/mm^3^ with 98% neutrophils, and the fluid culture was negative. The pigtail catheter was maintained for 7 days, with continued antibiotic treatment. Kidney allograft function and other laboratory parameters returned to baseline, and ciprofloxacin treatment was stopped after catheter removal.

## Discussion

In context of a clinical picture of an infection without a clear source in a transplant patient with a kidney cyst, cyst infection should be considered in the differential diagnosis. This diagnosis is infrequent, but can lead to serious illness and worsening kidney allograft function in the case of diagnostic delay (Table 2). With an increase in the age of living kidney donors because of more liberal donor inclusion criteria, one can expect to identify more simple cysts during predonation imaging screening.

**Table 2:** Teaching points

1.	Simple kidney cysts are not a contraindication to living donor kidney donation.
2.	Cyst unroofing or excision is generally not necessary but can be performed if the cyst is large and on the parenchymal surface.
3.	Previously unroofed kidney allograft cysts can regain full size due to allograft encapsulation.
4.	Recurrence of an unroofed cyst, in combination with abdominal pains and fever, point toward a cyst infection.
5.	The decision for an antibiotic regimen should be based on the ability to penetrate into the cyst cavity.
6.	Antibiotic therapy should be combined with cyst drainage.

Imaging studies showed an increase in the prevalence of simple cyst in the older population. Ultrasound screening of asymptomatic individuals, grouped as 30 to 49 years, 50 to 70 years, and more than 70 years of age revealed simple cysts in 1.7%, 11.5%, and 22.1%, respectively.S1 CT screening identified an even higher prevalence of 8.2%, 27.5%, 49%, and 60.6% in individuals 17 to 39 years, 40 to 59 years, 60 to 80 years, and more than 80 years, respectively.S2 Prevalence is higher in male individuals, individuals with a history of smoking, and patients with hypertension and chronic kidney disease.^4^^,^S3 In living kidney donor candidates, simple cyst prevalence ranges from 10% to 43%.^4^, ^5^, ^6^

Despite the high prevalence, simple cyst−related complications are rarely reported in native kidneys, and include flank pain, hematuria, intracystic hemorrhage, and pelvicalyceal obstruction in a small percentage of patients.S3 The most common symptoms requiring intervention for simple cysts are related to increase in cyst size and compression of the surrounding structures. Cyst aspiration, sclerotherapy obstruction, and laparoscopic excision are the interventions usually performed.

Experience with treating cyst infections derives from patients with autosomal dominant polycystic kidney disease (ADPKD), in which this complication is not uncommon. The preferred imaging modalities to diagnose cyst infection are magnetic resonance imaging and positron emission tomography–CT.S4 The choice of antibiotic for cyst infections should be based on the ability to penetrate into the cyst cavity. Quinolones are usually used as first-line treatment, but co-trimoxazole, chloramphenicol, and aminoglycosides may also provide adequate cyst penetration.S5 In addition to antibiotic treatment opportunities, drainage of the cyst under CT or ultrasound guidance is usually necessary.

In the case presented, the initial symptoms and subsequent laboratory findings did not raise suspicion for cyst infection during the patient’s first presentation. However, during the second admission, after 6 days, ultrasound findings pointed toward the diagnosis of kidney allograft cyst infection. This diagnosis was confirmed by the drainage of purulent fluid showing neutrophils. The treatment approach with a combination of antibiotic therapy and drainage resulted in prompt clinical improvement. The exact cause of the cyst infection was not determined, but we hypothesize that this could result from transient asymptomatic bacteremia; contrast-enhanced CT showed no connection of the cyst with the urinary tract.

To our knowledge, this is the first report of a simple kidney allograft cyst infection. If performed with the precautions outlined in international guidelines, transplantation of kidney allografts with simple, Bosniak grade 1, kidney cysts can be performed safely. However, cysts may recur despite unroofing and rarely present with infection.

## Disclosure

All the authors declared no competing interests.
