# Supplementary material for: Kidney Allograft Cyst Infection
Source: Kidney Int Rep. 2020 Apr 28;5(7):1114–7. doi: 10.1016/j.ekir.2020.04.013 (PMC7335975; doi:10.1016/j.ekir.2020.04.013)
Supplement: Supplementary File (PDF) [file mmc1.pdf]

Supplementary References

S1. Ravine D, Gibson RN, Donlan J, Sheffield LJ. An Ultrasound Renal Cyst Prevalence Survey: Specificity Data for Inherited Renal Cystic Diseases. Am J Kidney Dis. 1993;22(6):803-807. doi:10.1016/S0272-6386(12)70338-4

S2. Carrim ZI, Murchison JT. The prevalence of simple renal and hepatic cysts detected by spiral computed tomography. Clin Radiol. 2003;58(8):626-629. doi:10.1016/S0009-9260(03)00165-X

S3. Terada N, Arai Y, Kinukawa N, Yoshimura K, Terai A. Risk factors for renal cysts. BJU Int. 2004;93(9):1300-1302. doi:10.1111/j.1464-410X.2004.04844.x

S4. Sallée M, Rafat C, Zahar JR, et al. Cyst infections in patients with autosomal dominant polycystic kidney disease. Clin J Am Soc Nephrol. 2009;4(7):1183-1189. doi:10.2215/CJN.01870309

S5. Lantinga MA, Casteleijn NF, Geudens A, et al. Management of renal cyst infection in patients with autosomal dominant polycystic kidney disease: A systematic review. Nephrol Dial Transplant. 2017;32(1):144-150. doi:10.1093/ndt/gfv452
